# Chronic Hepatitis B in the Transplant Setting: A 30-Year Experience in a Single Tertiary Italian Center

**DOI:** 10.3390/v17040454

**Published:** 2025-03-21

**Authors:** Francesco Paolo Russo, Sara Battistella, Alberto Zanetto, Martina Gambato, Alberto Ferrarese, Giacomo Germani, Marco Senzolo, Claudia Mescoli, Salvatore Piano, Francesco Enrico D’Amico, Alessandro Vitale, Enrico Gringeri, Paolo Feltracco, Paolo Angeli, Umberto Cillo, Patrizia Burra

**Affiliations:** 1 Gastroenterology and Multivisceral Transplant Unit, Azienda Ospedale—Università di Padova, 35125 Padua, Italy; sara.battistella.4@studenti.unipd.it (S.B.); alberto.zanetto@unipd.it (A.Z.); martina.gambato@gmail.com (M.G.); alberto.ferrarese@unipd.it (A.F.); giacomo.germani@unipd.it (G.G.); marco.senzolo@aopd.veneto.it (M.S.); burra@unipd.it (P.B.); 2 Department of Surgery, Oncology and Gastroenterology, University of Padua, 35125 Padua, Italy; francescoenrico.damico@unipd.it (F.E.D.); alessandro.vitale@unipd.it (A.V.); enrico.gringeri@unipd.it (E.G.); cillo@unipd.it (U.C.); 3 Department of Medicine, (Pathology Section), University Hospital of Padua, 35125 Padua, Italy; claudia.mescoli@aopd.veneto.it; 4 Unit of Internal Medicine and Hepatology, Department of Medicine, University of Padua, 35125 Padua, Italy; salvatore.piano@unipd.it (S.P.); pangeli@unipd.it (P.A.); 5 General Surgery 2, Azienda Ospedale—Università di Padova, 35125 Padua, Italy; 6 Anaesthesia and Intensive Care, Department of Medicine, Padua University Hospital, 35125 Padua, Italy; paolo.feltracco@unipd.it

**Keywords:** HBV, HDV, liver transplantation, HBV prophylaxis, HBIG, NUCs

## Abstract

Background: Hepatitis B virus (HBV) remains a leading etiology for liver transplantation (LT). In a large cohort of HBsAg-positive patients, this study evaluates long-term patient and graft survival after LT over the past 30 years while analyzing trends and outcomes following waiting list (WL) inclusion over the last 15 years. Methods: HBsAg-positive patients who underwent transplantation between 1991 and 2020 and were waitlisted from 2006 to 2020 at Padua Hospital were included in the analysis. Patients were stratified according to hepatitis delta virus (HDV) coinfection, transplant indication (decompensated cirrhosis vs. hepatocellular carcinoma (HCC)), and WL inclusion period. Results: Among 321 HBsAg-positive LT recipients (31.5% HDV-coinfected, 46.4% HCC), 1-year and 5-year patient/graft survival rates were 87.6%/86.7% and 82.6%/82.2%, respectively. From 2006 to 2020, 284 HBsAg-positive patients were waitlisted (32.6% HDV-coinfected), with a significantly higher prevalence of HCC compared to non-HBV patients (*p* = 0.008). High-barrier nucleos(t)ide analogues (hbNUCs) significantly reduced mortality (*p* = 0.041) and improved survival post-WL inclusion (*p* = 0.007). Survival rates were consistent regardless of LT indication, HDV coinfection, or WL inclusion period. Post-transplant prophylaxis predominantly involved immunoglobulins (HBIG) + NUCs, resulting in only two cases of HBV reactivation, both clinically inconsequential. Conclusions: Over the past 30 years, HBV has remained a consistent indication for LT at our center. Thanks to hbNUCs, WL outcomes have improved and HCC has become the main indication for LT.

## 1. Introduction

Despite successful vaccination programs reducing hepatitis B virus (HBV) infections in younger populations and the advent of antiviral therapy altering disease progression, chronic hepatitis B (CHB) remains a significant indication for liver transplantation (LT) globally [1]. HBV infection leads to persistent viral replication, resulting in progressive liver damage, cirrhosis, and hepatocellular carcinoma (HCC) [2]. The five-year cumulative incidence of cirrhosis in untreated, chronically infected HBV patients ranges from 8% to 20% and 20% of cirrhotic patients eventually develop decompensated cirrhosis within five years [2]. Chronically infected patients have up to a 100-fold higher risk of developing HCC compared to non-infected individuals [3]. Indeed, HBV patients with cirrhosis face a 2–5% annual incidence of HCC [4]. Furthermore, CHB patients may develop HCC, even in the absence of cirrhosis, more frequently than those with other causes of liver disease [5]. HDV coinfection is the most severe form of viral hepatitis, leading to rapidly progressive liver disease and increasing the risk of decompensation and HCC [6,7].

The introduction and widespread application of high-barrier nucleos(t)ide analogues (hbNUCs) have significantly modified the natural history of HBV-related liver disease, reducing cirrhosis and HCC rates [8,9]. Jeong et al. [10] reported a 33.9% delisting rate within one year after the hbNUC introduction.

Although hbNUCs are highly effective, LT remains essential for CHB patients with end-stage liver disease or HCC within specific criteria [2]. Post-transplant prophylaxis, initially using immunoglobulins (HBIGs) alone and later combined with NUCs, has reduced HBV recurrence to less than 1%, ensuring graft and patient survival rates comparable to other liver disease etiologies [11,12,13].

In Western countries, HCV eradication [14], expanded HCC criteria [15], and the increasing prevalence of metabolic liver diseases [16,17,18] have significantly reshaped the liver transplant landscape [18]. Although the impact of metabolic-dysfunction-associated steatotic liver disease (MASLD) in Italy is mitigated by epidemiological and cultural factors, the current role of HBV in the LT setting remains unclear. The aims of our study were as follows:

- to assess overall long-term patient and graft survival in HBV patients who underwent a LT over the past 30 years;
- to analyze trends in waiting list (WL) inclusion and outcomes for HBV patients over the last 15 years in a tertiary center in Italy.

## 2. Materials and Methods

### 2.1. Data Collection and Study Design

All consecutive patients who underwent LT for HBV and/or HDV-related liver disease (defined by detectable HBsAg and anti-HDV positivity, respectively) at Padua University Hospital between 1991 and 2020 were included in this study. HCC developing in a non-cirrhotic liver, re-LT, age < 18 years, and acute liver failure (ALF) were exclusion criteria for this study. Non-cirrhotic HCC patients were excluded due to their limited number, which prevented statistical comparisons. ALF as an indication for LT was excluded as it represents a distinct clinical entity, subject to specific prioritization on the WL and urgent transplantation protocols. Data were collected on the age, gender, presence of HCC at LT, and HBV-related variables at the time of transplantation for all patients.

Patients were divided into two groups, based on their LT indication: the decompensated disease group (HBV-Dec), which included patients listed for decompensated cirrhosis (MELD ≥ 15 or MELD < 15 with MELD exceptions) with or without HCC, and the HCC group (HBV-HCC), which included patients listed with MELD < 15 and the presence of HCC.

Patient and graft survival were compared between the following groups:

- HBV and non-HBV patients;
- HBV-monoinfected and HDV-coinfected patients;
- HBV-Dec and HBV-HCC patients.

Furthermore, we performed a sub-analysis of patients included in the WL for LT from 2006 to 2020. Since 2006, all WL registrations had been prospectively recorded in an electronic database. For this subcohort, we performed a detailed analysis of liver function (Child–Turcotte–Pugh (CTP) and MELD score), pre- and post-LT antiviral therapy, body mass index (BMI), presence of portal vein thrombosis, refractory ascites, and patient outcomes (LT vs. exclusion from the WL vs. death vs. ongoing WL status).

To evaluate changes in WL inclusion indications and outcomes following the introduction and widespread use of hbNUCs at our center, we stratified WL patients into two time periods: 1. Patients registered between January 2006 and December 2013; 2. Patients registered between January 2014 and December 2020.

Trends in WL registration and outcomes were compared among:

- HBV and non-HBV patients;
- HBV-monoinfected and HDV-coinfected patients;
- HBV-Dec and HBV-HCC patients;
- Patients waitlisted between 2006–2013 and 2014–2020.

This study was conducted in accordance with the Declaration of Helsinki and all patients gave written informed consent at recruitment. The Padua University Hospital Ethical Committee approved this study (number: 3103/A0/14).

### 2.2. Patients’ Management After LT

Immunosuppressive (IS) therapy was administered according to the center’s protocol, in alignment with the European Association for the Study of the Liver (EASL) guidelines [19] and the recommendations of the International Liver Transplant Society (ILTS) consensus [20]. At our center, Basiliximab is commonly used during the induction phase to delay the introduction of calcineurin inhibitors (CNIs) and reduce the risk of acute rejection. It is administered twice, once during the anhepatic phase and again four days after surgery. Corticosteroids are typically prescribed during the first three months post-LT and are gradually tapered. Early withdrawal is preferred in case of metabolic comorbidities. CNIs remain the cornerstone of maintenance therapy. In the initial phase of our study, most patients received cyclosporine, which was later replaced by tacrolimus. CNI administration was individualized, particularly for patients with renal impairment and metabolic complications after LT. Cyclosporine blood levels (before administration C0) were maintained at 150–200 ng/mL during the first three months and then gradually reduced to approximately ~100 ng/mL. Tacrolimus blood levels were targeted at 6–10 ng/mL during the first three months, targeted around ~5 ng/mL from the fourth month to the end of the first year post-LT, and subsequently lowered to ~3 ng/mL. Adjunctive therapies included mycophenolate mofetil and azathioprine, used in selected cases (e.g., high-risk or prior episodes of acute rejection, autoimmune hepatitis, or to reduce the CNI dosage). Additionally, mTOR inhibitors, primarily everolimus, were predominantly used in patients who underwent LT for HCC as they have been shown to improve outcomes, particularly in individuals with high-risk tumors [21].

### 2.3. Statistical Analysis

Qualitative data are described using frequency and percentage. Quantitative data are described using a median with 25% and 75% quartile ranges. Comparisons between independent groups were performed using the Mann–Whitney U test, for continuous variables, and the Chi-square test or Fisher’s exact test, for categorical variables. Patient survival and graft survival were calculated from Kaplan–Meier curves and differences between patient groups were determined by the Log-Rank test. Statistical analysis was performed using IBM SPSS Statistics (Version 28). Statistical significance was set at *p* ≤ 0.05.

## 3. Results

### 3.1. Patients Undergoing Liver Transplantation Between 1991 and 2020

#### 3.1.1. Pre- and Post-Transplant Patients’ Characteristics

A total of 321 patients underwent transplantation for HBV-related liver disease at our center between 1991 and 2020; with 127 in the 1991–2005 cohort and 194 in the 2006–2020 cohort. Most patients were male (78.5%), with a median age at LT of 57 years (49.75–62). HCC was present in 46.4% of cases at LT. HDV coinfection was documented in 31.5% of patients with available data (254/321).

Due to the absence of an electronic medical record system before 2006 and the high number of transplants performed on patients from other Italian regions, post-transplant antiviral prophylaxis data were available only for 71.4% of patients. Most were treated with a life-long combination of NUCs and HBIG (90.7%) whereas 4.9% received NUC monotherapy and 4.4% received HBIG alone.

#### 3.1.2. Outcome After LT

**Figure 1:**
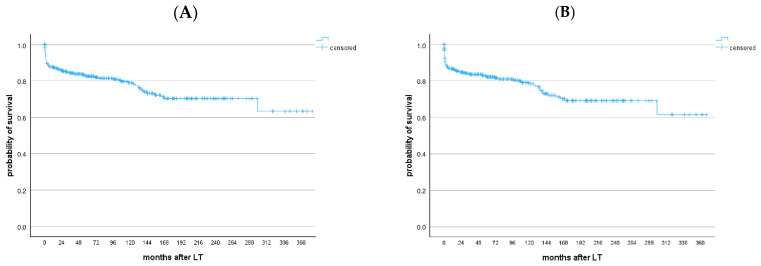
(**A**) Patient and (**B**) graft survival after the LT of HBV patients transplanted between 1991 and 2020. Patient survival and graft survival after LT were 87.6% and 86.7% at 1 year, 82.6% and 82.2% at 5 years, and 79% and 78.5% at 10 years, respectively.

Patient and graft survival after LT were 87.6% and 86.7% at 1 year, 82.6% and 82.2% at 5 years, 79% and 78.5% at 10 years, 70% and 69.2% at 20 years, and 63.3% and 61.5% at 30 years, respectively (Figure 1). Patients with and without HCC showed similar long-term patient survival (*p* = 0.797) (Figure S1). Similarly, no survival differences were observed between the 1991–2005 and 2006–2020 cohorts (*p* = 0.974 for patient survival, *p* = 0.961 for graft survival, Figure S2). HBV recurrence occurred in four patients (1.2%), two in each cohort, despite all patients receiving life-long HBIG + NUCs prophylaxis. None of those patients experienced clinically significant complications and all were alive at the end of the follow-up.

After a median follow-up time of 6.12 years (1.9–13.7), 21.7% of patients had died, 16.6% were lost to follow-up, and 61.8% remained alive. Sepsis was the leading cause of death after LT in both HCC (55.6%) and non-HCC patients (57.9%).

### 3.2. Analysis of the Waiting List Trends Between 2006 and 2020

**Figure 2:**
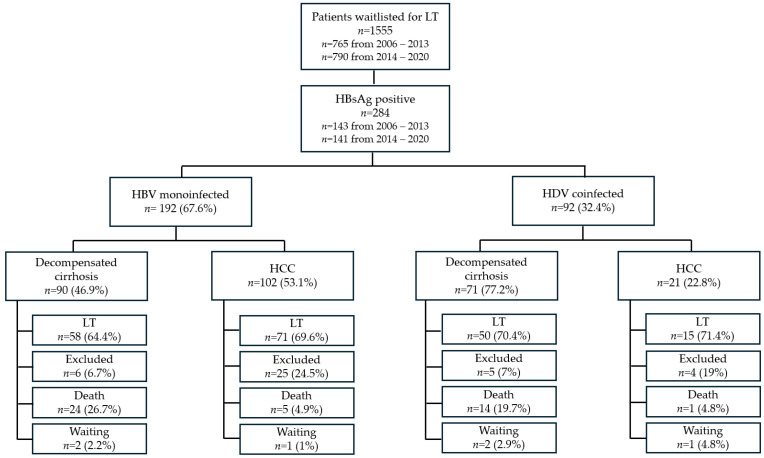
Patients waitlisted for LT from 2006 to 2020 and their outcomes after WL inclusion.

Since 2006, the implementation of an electronic medical record system at our center has enabled the collection of more precise data at the time of WL registrations and patient outcomes. Between January 2006 and December 2020, a total of 1555 patients were listed for LT at Padua Liver Transplantation Center. Most patients were male (76.6%), with a median age of 58 years (51–63) at WL registration time. Among them, 284 patients (18.2%) were HBsAg-positive, of whom 32.6% were coinfected with HDV. Decompensated liver disease was the main indication for WL registration (61.3%) while HCC was recorded in 50.9% of patients (Figure 2). Notably, HBV patients had a significantly higher prevalence of HCC (including those listed for decompensated cirrhosis) compared to non-HBV patients (58.1% vs. 49.3%, *p* = 0.008).

#### 3.2.1. Characteristics of HBV-Monoinfected Patients at WL Inclusion

HBV-monoinfected patients (*n* = 192) were predominantly male (85.9%), with a median age of 58 years (52.25–63) at WL registration. HCC was present in 67.2% of patients and was the main indication for LT for 53.1% of patients. Additionally, seven patients were diagnosed with HCC after WL inclusion. In patients listed for decompensated cirrhosis, the median MELD and Child–Turcotte–Pugh (CTP) scores at WL inclusion were 18 (15–24) and 10 (8–11), respectively. HBV DNA was detectable in 21.9% of cases. Antiviral therapy was administered to 89% of patients, mostly with ETV (42.7%). The baseline characteristics of HBV vs. non-HBV patients included in the WL are summarized in Table S1.

#### 3.2.2. Characteristics of HDV-Coinfected Patients at WL Inclusion

Among 92 HDV-coinfected patients, 57.6% were male, with a median age of 54 (47–58) years at WL registration. HCC was present in 39.1% of patients, with 12 additional cases diagnosed with HCC after WL inclusion. Decompensated cirrhosis was the predominant indication for LT (77.2%). In HDV-coinfected patients with decompensated cirrhosis, the median MELD and CTP scores at WL admission were 18 (15–23) and 9 (9–11), respectively. HBV DNA was detectable in 28.3% of cases. Antiviral therapy was administered in 93.5% of patients, primarily with ETV (51.1%). Compared to HBV-monoinfected patients, HDV-coinfected patients were more frequently female (*p* < 0.001), had a lower prevalence of HCC (*p* < 0.001), and were more frequently listed for decompensated cirrhosis (*p* < 0.001) Table 1.

#### 3.2.3. Trajectories of WL Inclusion

To analyze changes in WL inclusion and patient management, we divided the cohort into two groups: 1. Patients waitlisted between January 2006 and December 2013 (*n* = 765; HbsAg-positive *n* = 143); 2. Patients waitlisted between January 2014 and December 2020 (*n* = 790; HbsAg-positive *n* = 141).

**Figure 3:**
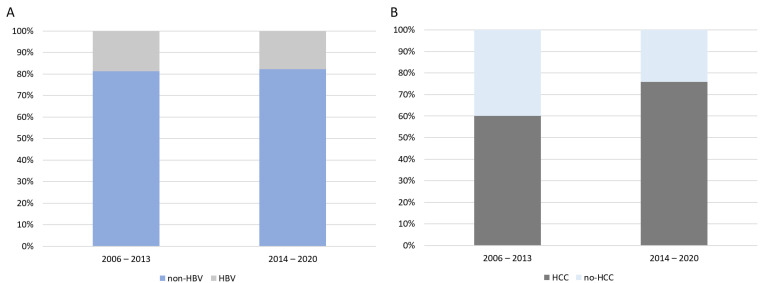
(**A**) Prevalence of HBV and non-HBV etiologies as indications for waiting-list inclusion over time; (**B**) Prevalence of HCC in HBV-monoinfected patients waitlisted for LT over time (*p* = 0.020).

During the 2006–2013 period, the majority of HBV patients received lamivudine as antiviral therapy while on the WL (LAM 40.1%, ETV 29.6%, no therapy 12.7%, TDF 12.7%, and ADV 4.9%). However, during the 2014–2020 period, the use of hbNUCs significantly increased compared to the first period (*p* < 0.001) (ETV 61.7%, TDF 27.7%, LAM 5%, no therapy 5.7%). The proportion of HBV patients on the WL remained stable over time (18.7% in the 2006–2013 period and 17.8% in the 2014–2020 period; *p* = 0.666) (Figure 3A). However, in HBV-monoinfected patients, the prevalence of HCC significantly increased, from 60% (2006–2013) to 75.9% (2014–2020) (*p* = 0.020), while it remained stable in HDV-coinfected patients (*p* = 0.355) (Figure 3B). Additionally, the prevalence of HDV coinfection significantly increased between the two periods (from 26.6% to 38.3%, *p* = 0.035). The median age at WL registration also increased (*p* = 0.013) (Figure S3), whereas the median MELD and CTP scores remained unchanged.

#### 3.2.4. The Impact of hbNUCs on Patients Waitlisted for LT

**Figure 4:**
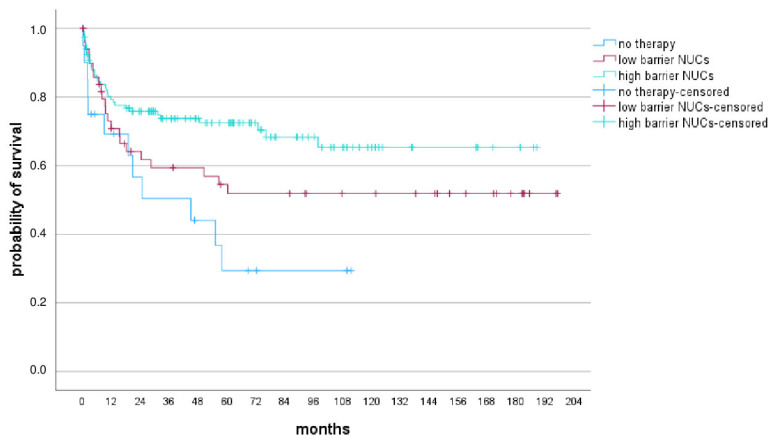
Patient survival after inclusion in the waiting list for LT according to antiviral treatment. Patients treated with high-barrier NUCs (ETV or TDF), represented by the green line, survived significantly longer after inclusion in the WL than patients treated with low-barrier NUCs (LAM, ADV), represented by the red line, or not treated with antiviral therapy, represented by the blue line; *p* = 0.007.

To better assess the impact of hbNUCs, patients were categorized into three groups: 1. Patients receiving lbNUCs, LAM or ADV (*n* = 71); 2. Patients who did not receive antiviral therapy (*n* = 26); 3. Patients receiving hbNUCs, ETV or TDF (*n* = 186). The characteristics of these three groups are summarized in Table 2. Compared to patients treated with lbNUCs or those who did not receive antiviral therapy, those receiving hbNUCs showed a lower WL mortality rate (26.9% vs. 19.7% vs. 12.4%, respectively), higher likelihood of undergoing transplantation (69.9% vs. 67.6% vs. 61.5%, respectively), and higher survival after WL inclusion (*p* = 0.007; Figure 4). However, no significant difference was observed in the delisting rate between the three groups (*p* = 0.664).

#### 3.2.5. Outcome of Patients After WL Inclusion

Patients with HBV-related liver disease had similar outcomes compared to non-HBV patients after WL inclusion, in terms of the probability of undergoing LT (68.3% vs. 60.7%, *p* = 0.2), de-listing rate (14.1% vs. 14.9%, *p* = 1), and death (15.5% vs. 21.9%, *p* = 0.073). At the end of the follow-up, six HBsAg-positive patients and 32 non-HBV patients were still on the WL. HBV patients had similar CTP and MELD scores at LT and comparable WL times compared to non-HBV patients.

#### 3.2.6. HBV-Monoinfected Patients

Among HBV-monoinfected patients waitlisted for decompensated cirrhosis (*n* = 90), 64.4% underwent LT, 26.7% died while on the WL, 6.7% were excluded, and two patients remained on the WL at the end of the follow-up. The median WL time was 112 (24–424.75) days.

Among patients waitlisted for HCC (*n* = 102), 69.6% underwent LT, 4.9% died while on the WL, 24.5% were excluded, and one was still waiting at the end of the follow-up (Figure 2).

The median time on the WL was 272 (125–636.50) days. HCC progression was the cause of WL exclusion for most patients (48%). For patients waitlisted for decompensated cirrhosis who underwent LT (*n* = 58), the median MELD and CTP scores at the time of the LT were 22 (16–30) and 10 (8–11), respectively.

#### 3.2.7. HDV-Coinfected Patients

Among HDV-coinfected patients waitlisted for decompensated cirrhosis (*n* = 71), 70.4% underwent LT, 19.7% died while on the WL, 7% were excluded, and two were still waiting at the end of the follow-up. The median WL time was 114 (46.5–337.5) days.

Among patients waitlisted for HCC (*n* = 21), 71.4% underwent LT, one died while on the WL, four were excluded, and one was still waiting at the end of the follow-up. The median time on the WL was 124.4 (88.75–320.25) days. HCC progression was the cause of WL exclusion in all patients (Figure 2).

For patients waitlisted for decompensated cirrhosis who underwent LT (*n* = 50), median MELD and CTP scores at LT were 22 (16.5–29.25) and 10 (9–11), respectively.

No significant differences were found between HDV-coinfected and HBV-monoinfected patients regarding MELD and CTP scores at LT, WL time, and WL outcomes (transplantation vs. death vs. exclusion vs. waiting).

#### 3.2.8. Long-Term Outcomes After LT

After transplantation, HBV recipients had patient and graft survivals of 86.1% and 85.1% at 1 year and of 80.6% and 80.2% at 5 years, respectively. Non-HBV recipients had patient and graft survivals of 86.1% and 85.4% at 1 year and 76% and 75.5% at 5 years, respectively. There were no significant differences in patient and graft survival between HBV and non-HBV patients (*p* = 0.131 and *p* = 0.152, respectively) (Figure S4).

#### 3.2.9. HBV-Monoinfected Patients

**Figure 5:**
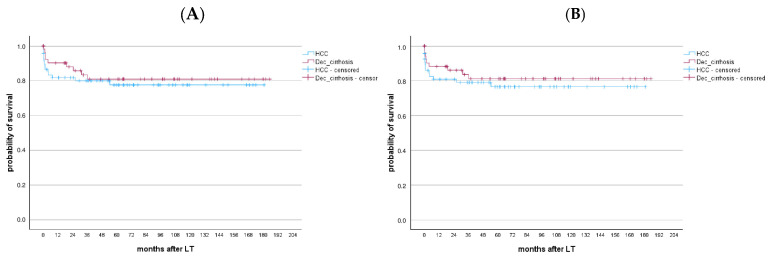
(**A**) Patient and (**B**) graft survival after the LT of HBV patients transplanted between 2006 and 2020 with HCC vs. no HCC. No differences in long-term patients and graft survival were found between HBV patients waitlisted for decompensated cirrhosis and HCC (*p* = 0.551 and *p* = 0.498, respectively).

Patient and graft survival rates after LT were 85.5% and 84.2% at 1 year and 79.2% and 78.8% at 5 years, respectively (Figure S5). Ten patients (7.8%) underwent re-transplantation, all within the first month post-LT. No significant differences in patient and graft survival were observed between HBV patients waitlisted for decompensated cirrhosis vs. HCC (*p* = 0.551 and *p* = 0.498, respectively) (Figure 5).

After LT, all patients, except for five individuals who received NUC monotherapy and one who received HBIG monotherapy, were administered combination therapy with life-long HBIG + NUCs, mostly with ETV (44.2%).

Only two patients (1.6%) experienced HBV reactivation after LT, with both receiving combination therapy (life-long HBIG + NUCs); however, no clinically significant complications occurred.

After a mean follow-up of 53.88 months (7.29–98.41), 17.8% of the patients died. The main cause of death after LT was sepsis, occurring in both patients without HCC (75%) and those with HCC (50%). Among patients with HCC, HCC recurrence was the cause of death in three patients (21.4%).

#### 3.2.10. HDV-Coinfected Patients

**Figure 6:**
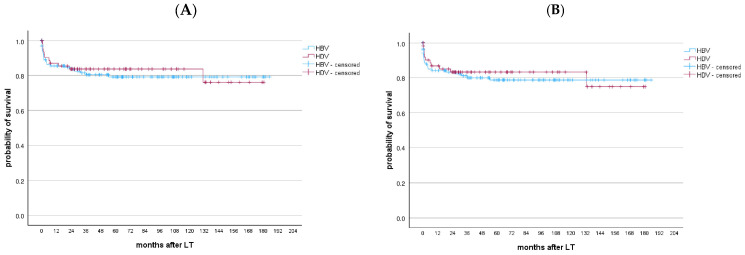
(**A**) Patient and (**B**) graft survival after the LT of HBV-monoinfected patients vs. HDV-coinfected patients transplanted between 2006 and 2020. No differences were found between HBV-monoinfected and HDV-coinfected patients in terms of patient and graft survival, *p* = 0.712 and *p* = 0.688, respectively.

The patient and graft survival after LT were 87% and 86.8% at 1 year and 83.7% and 83.3% at 5 years, respectively. No significant differences were found between HBV-monoinfected and HDV-coinfected patients in terms of patient and graft survival (*p* = 0.712 and *p* = 0.688, respectively), as shown in Figure 6. Similarly, no differences in long-term survival were observed between HDV-coinfected patients waitlisted for decompensated cirrhosis and those waitlisted for HCC (*p* = 0.829) (Figure S6). Seven patients (10.8%) required re-transplantation, with the majority (85.7%) occurring within the first month after LT. After LT, all patients, except for two individuals who received NUC monotherapy, received the combination therapy with life-long HBIG + NUCs, mainly ETV (56.9%). None of the HDV-coinfected patients experienced HBV recurrence after LT. After a median follow-up of 50.73 months (22.93–99.4), 16.9% of patients died, with sepsis being the leading cause of death (63.6%).

## 4. Discussion

This study highlights the evolving landscape of LT for CHB in Italy over the past 15 years. Despite advances in vaccination programs and new-generation antiviral therapies, CHB remained a stable indication for LT, accounting for 18.2% of WL admissions, similar to other Italian and European studies [1,22,23]. Migration patterns, particularly from sub-Saharan Africa and Eastern Europe during the past two decades, have influenced Southern Italy and may explain this trend [24,25,26]. Indeed, Padua University Hospital, located in the Veneto Region, North of Italy, ranks first in Italy in terms of the number of transplants performed on patients originating from other regions (46.6% of the total), especially from the south.

In the context of LT for HBV-related liver disease, our findings reveal a noteworthy shift: while overall LT numbers remained stable, there was an increase in cases of HCC and a decrease in decompensated cirrhosis cases. These results are similar to European data [1] and align with the introduction and accessibility of hbNUCs, which effectively halt the liver damage progression but do not completely eliminate HCC risk. The expanding indications for HCC and the possibility of considering more advanced tumors for LT may have contributed to these results [27]. Differences in demographics between HBV monoinfection and HDV coinfection patients arise from factors such as antiviral availability, vaccination programs, regional prevalence, migration patterns, and healthcare access. Migration, particularly from regions with a higher HDV prevalence, contributes to the observed variations in coinfection rates. In 2019, the overall prevalence of anti-HDV antibodies among HBsAg carriers in Italy was 9.9%; however, there was a substantial difference between native Italians (6.4%) and immigrants (26.4%) [28,29]. In our study, we found that patients on the WL who did not receive antiviral therapy experienced higher mortality, compared to those who were administered lbNUCs (LAM, ADV) and to those who received hbNUCs (ETV, TDF) (26.9% vs. 19.7% vs. 12.4%, respectively), and lower probability of undergoing transplantation (61.5% vs. 67.6% vs. 69.9%, respectively).

The introduction of hbNUCs was correlated with improved delisting rates from the WL [10]. In our cohort, we observed a significant improvement in survival rates after WL inclusion among patients who received hbNUCs compared to the patients who received lbNUCs or did not receive antiviral therapy (*p* = 0.007). However, delisting rates were similar between the three groups. Post-transplant outcomes were comparable between HBV patients and non-HBV patients, with patient survival and graft survival rates of 86.1% and 85.1% at 1 year and 80.6% and 80.2% at 5 years, respectively, vs. 86.1% and 85.4% at 1 year and 76% and 75.5% at 5 years, respectively. The leading causes of post-LT mortality were sepsis and HCC recurrence.

Antiviral prophylaxis predominantly involves the combination of life-long HBIG + NUCs, leading to only two cases of viral reactivation and characterized by very low viremia, without alteration of liver enzymes and/or any clinical consequences.

In recent years, different strategies have been proposed to reduce the dose of HBIG in favor of NUCs with increasing potency and decreasing resistance. Fung et al. [30] conducted a prospective study on 265 HBV-transplanted patients receiving ETV monotherapy, reporting no cases of detectable viral loads at 8 years and 85% overall survival at 9 years from LT. These results indicated that in low-risk patients (i.e., non-HIV, non-HCC, non-HDV, undetectable HBV DNA at LT, and good adherence to clinical and biochemical follow-up), HBIG-free prophylaxis with hbNUCs may be a safe and cost-effective strategy. However, further randomized studies are needed to better understand the effect of HBIG-free prophylactic regimens on liver reinfection and its clinical consequences [31]. This study has several limitations. First, the retrospective nature of this study introduces selection bias and makes it impossible to establish the temporal relationship and the causality between events. Additionally, the electronic medical record system was introduced in our center only in 2006, meaning we could not collect adjunctive data on liver function and the type of antiviral therapy administered before and after LT for patients who underwent LT before 2006. Another limitation of this study is the absence of HDV-RNA assessment, which prevents us from confirming that the patients identified as HDV-positive truly exhibited detectable viremia. Furthermore, nearly half of all transplant procedures conducted at our center are performed on patients originating from other regions within Italy. Subsequently, these recipients receive follow-up care at their local hepatological center. This study is characterized as a monocenter investigation, which, while potentially limiting its generalizability to broader populations, offers notable strengths stemming from the extensive, detailed experience within a single center utilizing consistent practices and protocols for immunosuppression and antiviral management over consecutive time periods. The benefits of observing changes in NUCs and the management of HCC cases by the same team are particularly noteworthy. Such observations provide valuable insights into the independent impacts on outcomes, including long-term graft and patient survival among HBV patients, as well as significant shifts in the composition of patients within the transplant waiting list.

Future prospective studies are warranted to delve deeper into the optimal antiviral prophylactic strategies post-LT and to refine the characterization of patients who may potentially benefit from HBIG-free prophylaxis.

## 5. Conclusions

End-stage liver disease due to chronic hepatitis B remains a prevalent indication for LT. The introduction and widespread use of hbNUCs have led to substantial changes in the natural progression of liver disease before and after LT. These advances have, on the one hand, led to a remarkable decrease in mortality among patients on the WL for LT and, on the other hand, caused a shift in the main indication for LT, from decompensated cirrhosis to HCC. After transplantation, individuals with CHB exhibited similar patient and graft survival rates compared to those suffering from other etiologies of liver disease. The gold standard for prophylactic management is the combination therapy of life-long HBIG and hbNUCs, especially for managing high-risk patients. Prospective studies are urgently needed to assess the best antiviral prophylaxis for HBV patients after transplantation.

## Figures and Tables

**Table 1:** Characteristics of HBV-monoinfected and HDV-coinfected patients at waiting list inclusion.

Variables at WL Inclusion	HBV-Monoinfected Patients (*n* = 192) N (%), Median (IQR)	HDV-Coinfected Patients (*n* = 92) N (%), Median (IQR)	*p* Value
Sex, male	(85.9)	(57.6)	<0.001
Age	58 (52.25–63)	54 (47–58)	<0.001
Indication for LT - HCC - Decompensated cirrhosis			<0.001
	102 (53.1)	21 (22.8)	
	90 (46.9)	71 (77.2)	
HCC, yes	129 (67.2)	36 (39.1)	<0.001
Portal vein thrombosis	30 (15.6)	8 (8.7)	0.223
Refractory ascites	32 (16.7)	22 (23.9)	0.145
MELD (Dec_cirrhosis)	18 (15–24)	18 (15–23)	0.485
CTP (Dec_cirrhosis)	10 (8–11)	9 (9–11)	0.905
HBV DNA detectable	42 (21.9%)	26 (28.3)	0.977
Antiviral therapy before LT, yes - LAM - ETV - TDF - Other	171 (89)	86 (93.5)	0.249
	44 (22.9)	20 (21.7)	
	82 (42.7)	47 (51.1)	
	38 (19.8)	19 (20.7)	
	7 (3.7)	0 (0)	

**Table 2:** Characteristics of patients according to the type of antiviral therapy before liver transplantation.

Variables at WL Inclusion	No Therapy (*n* = 26) N (%), Median (IQR)	lb-NUCs (*n* = 71) N (%), Median (IQR)	hb-NUCs (186) N (%), Median (IQR)	*p* Value
Sex, male	22 (84.6)	56 (78.9)	139 (74.7)	0.472
Age	56.5 (48.75–63.25)	55 (48–60)	57 (51–62)	0.405
HDV coinfection	6 (23.1)	20 (28.2)	66 (35.5)	0.299
HBV DNA detectable	8 (30.8)	15 (21.1)	45 (24.3)	0.613
Indication for LT - HCC - Decompensated cirrhosis	8 (30.8)	30 (42.3)	84 (45.2)	0.376
	18 (69.2)	41 (57.7)	102 (54.8)	
HCC, yes	10 (38.5)	41 (57.7)	113 (60.8)	0.098
Portal vein thrombosis	2 (7.7)	10 (14.1)	26 (14)	0.139
Refractory ascites	5 (19.2)	16 (22.5)	33 (17.7)	0.682
MELD (Dec_cirrhosis)	19 (17.25–28.5)	16 (13–21)	19 (16–24)	0.043
CTP (Dec_cirrhosis)	9 (8–11.25)	9 (8–11)	10 (9–11)	0.743
Outcome in WL - LT - Death - Excluded - Waiting				0.255
	16 (61.5)	48 (67.6)	130 (69.9)	
	7 (26.9)	14 (19.7)	23 (12.4)	
	3 (11.5)	9 (12.7)	27 (14.5)	
	-	-	6 (3.2)	

## Data Availability

The data presented in this study are available upon reasonable request from the corresponding author (F.P.R.).

## Supplementary Materials

The following supporting information can be downloaded at: https://www.mdpi.com/article/10.3390/v17040454/s1, Figure S1: Patient survival after LT of HBV patients transplanted between 1991 and 2020 without HCC vs. with HCC; Figure S2: (A) Patient and (B) graft survival after the LT of HBV patients transplanted between 1991 and 2005 vs. patients transplanted between 2006 and 2020; Figure S3: Age at WL inclusion between 2006 and 2013 vs. 2014 and 2020. Median age increases over time, from 55 (50–61) in the 2006–2013 period to 57 (51–63) in the 2014–2020 period, *p* = 0.013; Figure S4: (A) Patient and (B) graft survival after the LT of HBV vs. non-HBV patients transplanted between 2006 and 2020; Figure S5: (A) Patient and (B) graft survival after the LT of HBV patients transplanted between 2006 and 2020; Figure S6: Patient survival after the LT of HDV-coinfected patients transplanted between 2006 and 2020 for HCC vs. those transplanted for decompensated cirrhosis; Table S1: Characteristics of HBV vs. non-HBV patients at waiting list inclusion.

## Abbreviations

The following abbreviations are used in this manuscript:

HBV	hepatitis B virus
HDV	hepatitis delta virus
CHB	chronic hepatitis B
LT	liver transplantation
HCC	hepatocellular carcinoma
NUCs	nucleos(t)ide analogues
hbNUCs	high-barrier nucleos(t)ide analogues
lbNUCs	low-barrier nucleos(t)ide analogues
WL	waiting list
HBV-Dec	HBV-related decompensated cirrhosis
HBV-HCC	HBV-related hepatocellular carcinoma
MELD	model for end-stage liver disease score
CTP	Child–Turcotte–Pugh score
BMI	body mass index
IS	immunosuppressive
IQR	interquartile ranges
LAM	lamivudine
ETV	entecavir
ADV	adefovir
TAF	tenofovir alafenamide
TDF	tenofovir disoproxil
