# Supplementary material for: Chronic Hepatitis B in the Transplant Setting: A 30-Year Experience in a Single Tertiary Italian Center
Source: Viruses. 2025 Mar 21;17(4):454. doi: 10.3390/v17040454 (PMC12030929; doi:10.3390/v17040454)
Supplement: Supplementary file 1 [file viruses-17-00454-s001.zip › viruses-3484573-supplementary.pdf]

Supplementary Material

Figure S1. Patient survival after LT of HBV patients transplanted between 1991-2020 without HCC vs. with HCC.

Patients with and without HCC showed similar long-term patient survival (p=0.797).

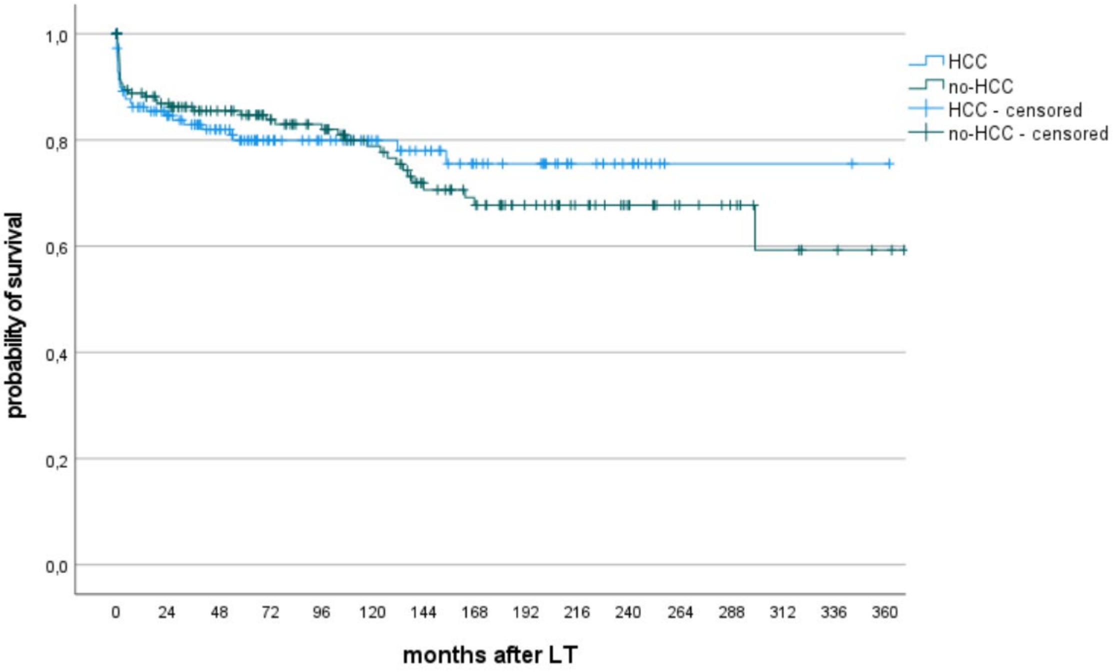

Figure S2. (A) Patient and (B) graft survival after LT of HBV patients transplanted between 1991-2005 vs patients transplanted between 2006 and 2020.
No difference in patient and graft survival was found between the cohort of patients who underwent transplantation between 1991 and 2005 and those who underwent transplantation between 2006 and 2020 (p=0.974 and p=0.961, respectively).

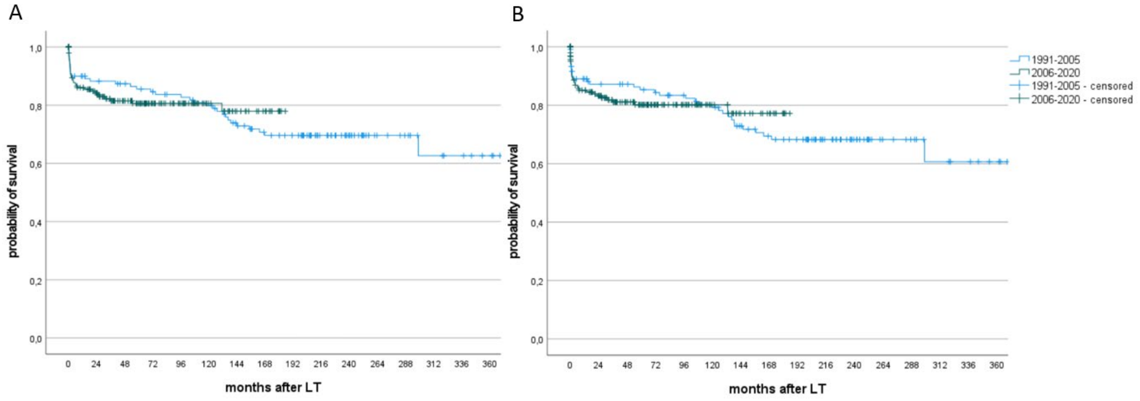

Figure S3. Age at WL inclusion between 2006-2013 vs. 2014-2020. Median age increases over time, from 55 (50 – 61) in 2006-2013 to 57 (51 – 63) in 2014-2020, p=0.013.

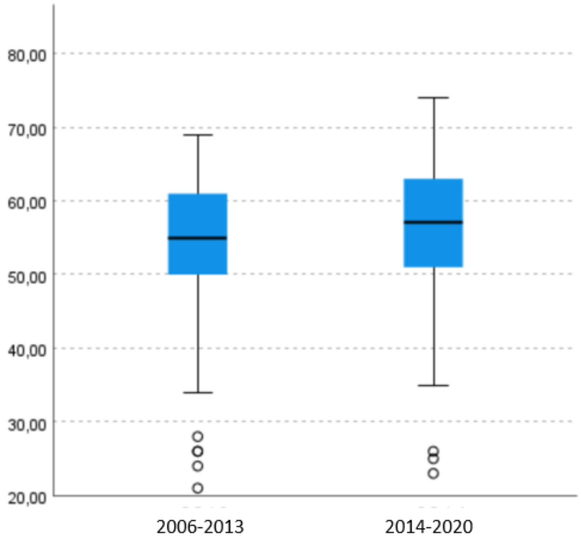

Figure S4. (A) Patient and (B) graft survival after LT of HBV vs. non-HBV patients transplanted between 2006-2020.

Patient and graft survival between HBV and non-HBV patients was not significant different (p=0.131 and p=0.152, respectively)

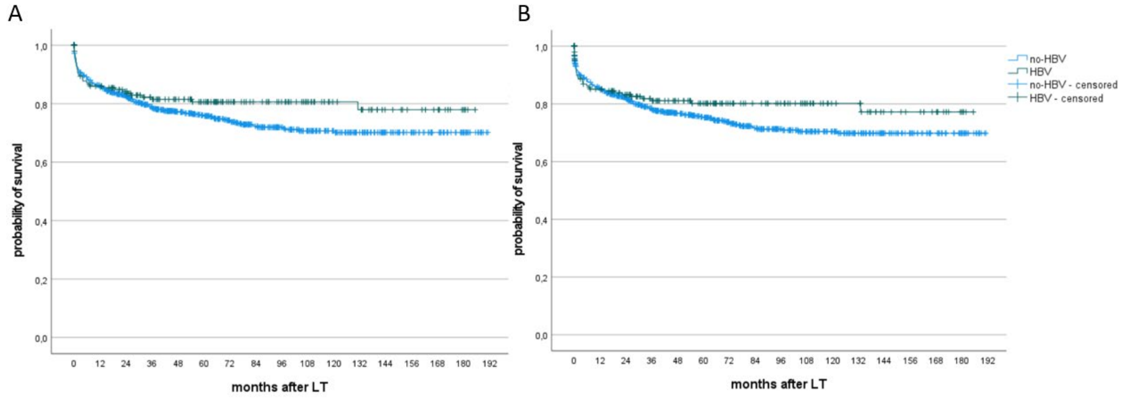

Figure S5. (A) Patient and (B) graft survival after LT of HBV patients transplanted between 2006-2020.

Patient and graft survival after LT were 85.5% and 84.2% at 1-year and 79.2% and 78.8% at 5-years, respectively.

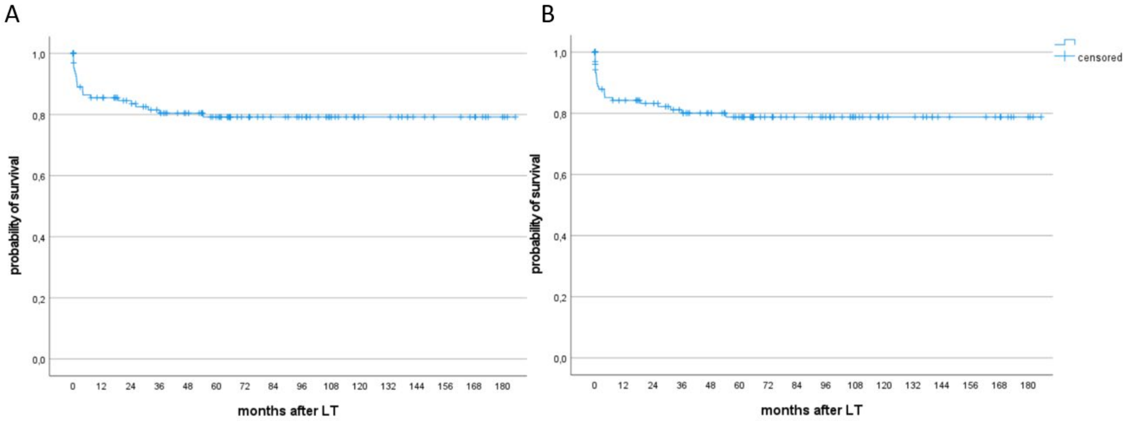

Figure S6. Patient survival after LT of HDV coinfected patients transplanted between 2006-2020 for HCC vs. those transplanted for decompensated cirrhosis.

No differences in long-term survival were found between HDV coinfected patients waitlisted for decompensated cirrhosis and HCC (p=0.829).

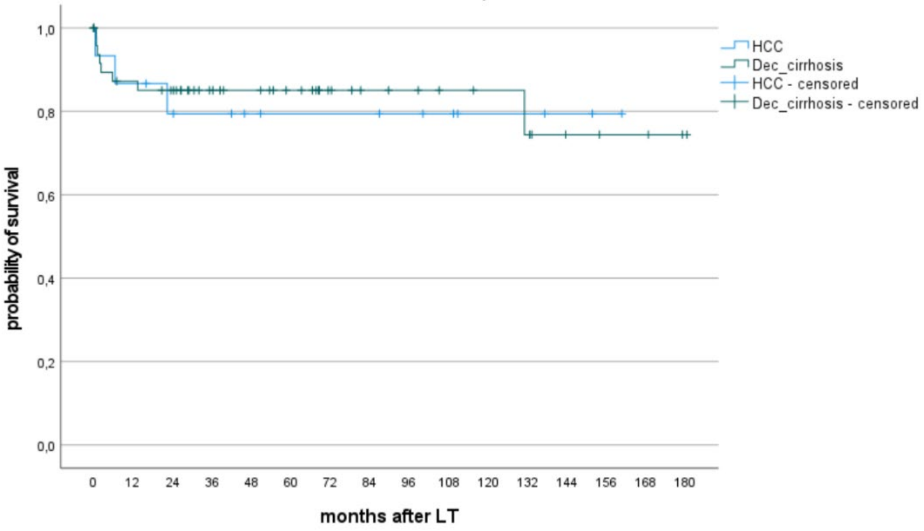

Table S1. Characteristics of HBV vs. non-HBV patients at waiting list inclusion.

Variables at WL inclusion	HBV patients (n=284) N (%), median (IQR)	Non-HBV patients (n=1271) N (%), median (IQR)	P value
**Sex, M**	218 (76.8)	973 (76.6)	0.941
**Age**	56 (50 – 62)	58 (51 – 63)	0.757
**MELD (Dec_cirrhosis)**	14 (9.25 – 19)	15 (10 – 19)	0.233
**CHILD (Dec_cirrhosis)**	8 (6 – 10)	9 (6 – 10)	0.916
**Indication for LT, decompensated cirrhosis**	161 (56.7)	792 (62.3)	0.079
**HCC, yes**	165 (58.1)	627 (49.3)	0.008
**Refractory ascites**	315 (24.8)	54 (19)	0.039
**PVT**	193 (15.2)	38 (13.4)	0.547
**Time on WL, days**	178 (58 – 468.75)	209 (71 – 484)	0.820
